# Caregiver and child question types during a museum interaction

**DOI:** 10.3389/fpsyg.2024.1401772

**Published:** 2024-07-09

**Authors:** Jill C. Thorson, Jill M. Trumbell, Kimberly Nesbitt

**Affiliations:** ^1^Department of Communication Sciences and Disorders, University of New Hampshire, Durham, NH, United States; ^2^Department of Human Development and Family Studies, University of New Hampshire, Durham, NH, United States

**Keywords:** question forms, child language, caregiver–child interactions, children’s museum, language development

## Abstract

Children museums provide an engaging learning environment for families with exhibits designed to stimulate caregiver-child interactions. Specific types of questions have been shown to support child language learning by scaffolding more elaborative responses. This study analyzed the use of question form types during caregiver-child interactions in a children’s museum, aiming to discern their correlation with child language proficiency. We examined and transcribed two exhibit explorations by 43 caregiver-child dyads (3- to 6-year-old children). Our analysis encompasses various syntactic question types (e.g., yes-no, wh-) and measures of child language proficiency, including lexical diversity, morphosyntactic complexity, and overall language ability. Findings reveal disparities in question form usage among caregivers and children, with caregivers predominantly employing closed questions and children balancing closed and open-ended types. Children of caregivers who predominantly posed closed questions exhibited shorter utterances and lower overall language scores. Details on other question forms are presented (sub-types of polar, wh-, alternative, and echo). These findings contribute to our understanding of how question form influences language development and caregiver–child interactions.

## 1 Introduction

Children’s museums provide dynamic environments for children and families to engage in learning as well as ecologically valid locations to observe natural caregiver-child interactions. Exhibits are designed to enhance and stimulate these exchanges with research suggesting that caregiver behavior directly influences the content of caregiver-child interactions and children’s cognitive outcomes (Marcus, 2016; Willard et al., 2019; Callanan et al., 2020). Willard et al. (2019) showed that caregiver encouragement and exploration predicted child discussions and interaction with the exhibit topic. Caregiver questions predicted the amount of time that the children spent on follow-up tasks in the museum, engaging with material for more time and demonstrating a link between increased question-asking and positive outcomes in this setting. When the caregiver was specifically encouraged to explain things to the child, this led to greater discussions of topics relevant to the exhibit. Relatedly, specific types of parent questions have been shown to support narrative development and critical thinking among young children by scaffolding more elaborative responses in a home play environment (Reese and Fivush, 1993). Overall, adult-child interactions provide opportunities for children to practice language, engage in and learn conversational rules, and hear advanced linguistic models from the adult (Bohannon and Bonvillian, 1997). The current study explores specific question forms as linguistic models used by caregivers during museum exploration, how these patterns are reflected in the question forms produced by children, and potential relationships between caregiver question form types and child language. Specifically, this study offers a natural setting to observe authentic caregiver-child interactions and the use of different question forms.

Due to their history of providing interactive learning settings and supporting social interactions, museums have been the focus of a body of learning research since the early 2000s. A 2017 review of the past decade revealed that more than two-thirds of museum research has been conducted in science and history museums, with much less (14%) in children’s museums (Andre et al., 2017). Broadly, these observational studies in museums show that when children and caregivers are engaged in hands-on learning activities, their conversations during these interactions are related to the overall quality of their engagements in the exhibits (Gleason and Schauble, 1999; Crowley et al., 2001a,b). However, most work has focused on caregiver-child interactions with school-aged children, with the average age of participants around 9 years old in these studies (Andre et al., 2017). Fewer studies have looked at caregiver-child interactions in children’s museums among samples of preschool-aged children and their parents, a developmental time frame of interest given children’s rapidly expanding language acquisition during this time (Andre et al., 2017). The current study fills this gap by providing data from parent–child exchanges in a children’s museum for children between the ages of 3 and 6 years and specifically their use of various question forms.

Questions may be broken down into a variety of categories and types, with the form and function of questions to children impacting learning in distinct ways. The *form* of a question refers to how it is syntactically structured (e.g., wh-questions vs. yes-no questions). Within these broad forms, there are also sub-types of different varieties of syntactic structures (e.g., an inverted yes-no question vs. a tag question vs. a wh-question). Models such as Leach’s 15 interrogative types classified adult questions based on their syntactic constraints and has been applied to maternal speech to analyze patterns (Leach, 1972; Toler and Bankson, 1976). Research has also developed coding schemes to identify question-response sequences by their semantic status (polar, content, or alternative) and applied this division to natural conversation corpora (Stivers, 2010; Stivers and Enfield, 2010; Sadock, 2012). On the other hand, the *form* of a question may be distinct from its *function,* or its speech act (Grice, 1975; Searle et al., 1980; De Ruiter, 2012). Questions may serve different *functions* or social actions such as pedagogical questions, information-seeking, confirmation-seeking, or rhetorical (Stivers and Enfield, 2010; De Ruiter, 2012; Yu et al., 2018, 2019) even when the form may relay something distinct. For example, what is formally a question may functionally be a statement or request, or vice versa. Along with *function*, research on child cognitive development in children’s museums has coded for the causal nature of language for both statements and questions, classifying how questions result in exploring causation (vs. their syntactic form) (Callanan et al., 2020). For the current study, the overarching goal is to quantify and analyze the *form* of questions that caregivers used with their children versus the *function* of those questions. As an in-depth analysis of question type has not yet been conducted in a children’s museum with this age range, the *form* of questions was selected as a first step in understanding the types of questions that both caregivers and children use in this setting. Here, we start broadly by analyzing *form* and reviewing how caregivers and children construct their questions, expanding our classification system to capture sub-types of questions that incorporate semantics (e.g., polar declarative questions and echo questions that both use prosody to indicate the question nature of the utterance). Future work plans to expand this analysis into the functional domain and connect *form* and *function* to the prosodic patterns employed (Geluykens, 1988).

A subset of studies at museums have analyzed ways that language may support learning and engage children in the exhibit, art, or activity. Caregiver scaffolding behaviors that have been suggested to support this type of learning, specifically in the case of art galleries/museums, include providing explanations, making suggestions, and asking open-ended questions (Piscitelli and Weier, 2002; Weier, 2004; Benjamin et al., 2010; Andre et al., 2017). Of interest to the current study is the use of questions, which can be broken down into two broad categories: open-ended versus closed questions. Open-ended questions have multiple possible answers without constraint (Hargreaves, 1984; de Rivera et al., 2005). For example, wh-questions (i.e., content or Q-word questions; questions that begin with *who, what, why, when, where,* or *how*; Stivers and Enfield, 2010) generally provide an opportunity for longer responses and increased engagement in the interaction (e.g., *What is happening over there? The king is building a castle to live in*.). In contrast, closed questions (or forced choice questions) restrict the number of potential responses to one. For example, a yes-no question (or polar question) semantically seeks confirmation/affirmation or disconfirmation and intends to limit the responder to answer with either ‘yes’ or ‘no’ (e.g., *Do you want to play? Yes*.). Sub-types of the polar question include its interrogative form (with auxiliary inversion), declarative form (without auxiliary inversion; indicated with prosody), and tags (*You want to go, yes?*), all of which limit the responder (Stivers and Enfield, 2010). Another type of closed question is an alternative question (either/or questions), where the response is limited to one of the options presented in the question (e.g., *Do you want to play with the animals or the blocks? The blocks.*). Formally defining these structures is one approach to understanding the complexity of questioning during development, while also recognizing the need to incorporate how questions are used functionally and socially in this framework [see De Ruiter (2012b) for a more comprehensive review and discussion on the interaction of form and function in questioning].

Open-ended versus closed question categories may be implemented differently across fields. If differentiating the categories in terms of the pragmatics and not only syntax-semantics, there are cases where wh-questions may behave similarly to closed questions (e.g., *What is that in your hand?* where the interested response is specified with the narrow focus nature of the question). This type of more constrained or narrow focus response is more likely with *who*, *when*, *where*, and *what*. In Stivers and Enfield (2010), their definition of Q-word questions focuses on wh-questions where a part of the question is presupposed and the question seeks the “identity of one element of the presupposition” (p. 2621). In contrast, the same wh- words may also be used in broad focus which allows for greater leniency in the response (e.g., *What are you doing today?, Who do you think is going to be in there?*) (Kuchirko et al., 2016). For *why* and *how*, these two types typically elicit a more extended response and may not restrict the answer in the same way as the other wh-question types. In the current study, we include all wh-questions in the open-ended category as we employ a more syntactic form approach here. Also, wh-questions have been shown to promote language development and thus seem to hold an distinct role during social interactions versus closed/forced choice questions (Rowe et al., 2017). Future work would benefit from dividing these categories in different manners, including in a functional pragmatic way that considers visual information from eye gaze and/or head-nods, which may additionally alter the function of the utterance (De Ruiter, 2012a). These two broad categories of questions and their sub-types have been the focus of research in museum settings as well as in the home and classroom.

Questions have been shown to play a foundational role in language development and learning (Fletcher et al., 2008; Blewitt et al., 2009; Kuchirko et al., 2016; Yu et al., 2018). Work analyzing the use of questions to elicit responses suggests that more open-ended wh-questions support engagement and learning (Borun et al., 1997; Haden, 2010; Cristofaro and Tamis-LeMonda, 2012; Rowe et al., 2017). Haden (2010) analyzed how elaborative conversation aids scientific concept learning, with open-ended wh-questions and explanatory comments playing a key role in generating that type of exchange. Based on this work, Haden et al. (2014) provided conversation instruction to adults during STEM activities in a children’s museum with 4- to 8-year-old children, resulting in doubling the number of wh-questions produced by families receiving those instructions. Benjamin et al. (2010) also showed that the use of open-ended wh-questions is an effective way to facilitate understanding between caregivers and their children in children’s museums. Finally, Rowe et al. (2017) further associated wh-question use with larger vocabularies and increased verbal reasoning scores for 2-year-olds due to their ability to elicit more frequent and grammatically complex responses. Thus, the quality and type of language use has been shown to be as critical as the quantity of language exposure a child receives (Hart and Risley, 1995; Rowe, 2012; Weisleder and Fernald, 2013; Hirsh-Pasek et al., 2015; Rowe et al., 2017; Rowe and Snow, 2020).

Parental questions directed to young children have often been explored via maternal language. Early work by Newport et al. (1977) examined maternal speech styles of 14 mothers of 12- to 27-month-old children, analyzing the frequency of sentence types including yes-no and wh-questions. Declaratives (statements of information) made up only 30% of the mother’s utterances to their children versus 80% of their utterances when speaking to the experimenter (i.e., adult-directed speech). The remainder of the utterances to the children were imperatives or commands (18%), wh-questions (15%), yes-no questions (21%), and deictic questions to clarify time, location, or person (8%), with yes-no questions being the most frequent question type after declaratives. Relating to child language measures, this study and follow-up work showed that the use of yes-no questions positively correlated with acquiring auxiliary verbs, but only in the later age range of 24–27 months old (Newport et al., 1977; Gleitman et al., 1984). Barnes et al. (2012) replicated Newport et al. (1977) and Gleitman et al. (1984) by breaking down question types further by including inverted yes-no questions (e.g., *do you want something to eat?*), non-inverted yes-no questions (e.g., *you do not want anything to eat?*), and tag questions (e.g., *you want something to eat, right*?). They showed that it was inverted yes-no questions that had the strongest connection to auxiliary verb gains and grammatical development. Taken together, this early work highlights the importance of question use to support language learning and the role that different types of question forms can have in not only generating responses from children but also modeling grammatical forms.

Research has examined child response rates to different types of questions, with closed questions more likely to elicit a response from the child (regardless of utterance length) and open- ended questions often resulting in longer responses. For example, Olsen-Fulero and Conforti (1983) showed that 2.5- to 3-year-old children were more likely to respond to their mother’s closed versus open-ended questions. This could potentially be due to the younger age of the children, where the closed questions require a simpler response. In the case of wh-questions, increasing the number of wh-questions in the caregiver input to a 2-year-old correlated with an increase in later vocabulary development and skill due to the increased complexity needed for responding (Rowe et al., 2017; Rowe and Snow, 2020). For preschoolers aged 2–5 years old, children who asked information-seeking ‘how’ and ‘why’ questions, were more likely to follow up with another question to keep the conversation moving forward (if the caregiver had responded; Frazier et al., 2009). This aligns with work showing that questions, particularly open-ended wh-questions, are helpful for language learning for toddlers in areas such as verb learning and wh-question acquisition (e.g., Hoff-Ginsberg, 1986; Rowland et al., 2003; Valian and Casey, 2003). Beyond maternal language, fathers have been found to ask more wh-questions than mothers (Gleason, 1975; Tomasello et al., 1990; Rowe et al., 2004), with work showing that fathers’ wh-questions stimulated toddler (2-year-olds) responses with a longer mean length of utterance (MLU) than other types of questions (Rowe et al., 2017). Overall, wh-questions can motivate an extended conversation, providing the opportunity for increased language input and fostering verbal reasoning.

A considerable amount of research has been dedicated to how teachers use questions in a classroom setting. While this is a distinct learning environment from a museum or the home with a caregiver—with research showing that children behave differently in terms of question asking with their parents than with their teachers (Fitneva, 2012)—this body of work lends insight into the types of questions utilized and their impact. Like with caregivers, open-ended question use by teachers in a classroom setting has been linked to improvements in cognitive skills since these questions encourage individuals to verbalize and elaborate upon their responses (Çakır, 2016). Through elaboration, opportunities to use language also increase. Harlen (1999) showed that open-ended questions also helped build vocabulary through the encouragement of expanded responses. In a study with 3-year-old preschoolers, high (open-ended) and low (closed) constraint teacher questions both successfully elicited child responses (Honig and Wittmer, 1982; Wittmer and Honig, 1991). Though both types led to a response from the child, the authors suggested that teachers use more open-ended questions to support cognitive and linguistic complexity. Similarly, Wood and Wood (1983) conducted a preschool study with 3- to 4-year-old children where they also found that open-ended questions elicited longer and more elaborative responses than closed questions. Interestingly, the more questions the teacher asked in this study, the less likely children were to show initiative, elaborate, follow up with additional information, or respond at all. This led to their suggestion to decrease the amount of questioning in a preschool setting. While overall the literature supports the use of open-ended questions by teachers, this type of finding raises the question of whether open-ended question usage is impacted by, or interacts with, the overall amount of questioning in this setting and whether similar findings can be found in other learning settings.

De Rivera et al. (2005) studied the use of specific question types by 13 preschool teachers and 13 teachers of toddlers. In addition to open-ended and closed questions, this study also included topic-continuing versus topic-initiating questions, which classify questions based on whether the topic was continued or if a new one began, regardless of the syntactic form. While they found that the educators’ pattern of usage did not differ by age, the effects on child responses varied. The toddler group (~2 years old) showed little effect of teacher question type, but the preschooler group (~4 years old) showed more multiword utterance usage when the teacher used open-ended or topic-continuing questions (de Rivera et al., 2005). Overall, for educators of young children, fewer than 50% of their sentence types are questions and the majority of these questions were of the closed type (Schaffer and Liddell, 1984; Wittmer and Honig, 1991; O’Brien and Bi, 1995; de Rivera et al., 2005) with related work finding that up to 92% of a teacher’s questions may in fact be closed (Bay and Hartman, 2015).

In the current study, we have three research aims to examine the use of different question forms by caregivers and children during exhibit exploration in a children’s museum. The first two aims are descriptive and seek to understand the range of question types utilized in a children’s museum setting between a caregiver and a child. The first research question asks what types of questions caregivers use when exploring a museum exhibit with their child. Research in children’s museums shows that these settings are designed as natural learning environments that encourage direct, hands-on learning in facilities with open spaces, realistic exhibits, and exhibits that evoke emotional responses (Lewin, 1989). Past work suggests that when caregivers more actively engage with their children in exhibits, children will spend more time and learn more in that setting (Puchner et al., 2001). Specifically, caregivers who use scaffolding in this situation will support learning through conversation. Thus, we hypothesize that caregivers will use a range of syntactic question types, with the prediction that while there will be variation amongst caregivers in their ratio of open-ended (wh-) to closed-ended questions, they will produce more closed-question types than open-ended questions (Newport et al., 1977; Schaffer and Liddell, 1984; Wittmer and Honig, 1991; O’Brien and Bi, 1995; de Rivera et al., 2005; Bay and Hartman, 2015). Additionally, as this study will focus on the form of questions, we have broken down question types into *form* categories based on past work in interrogative models on syntax and semantics (Leach, 1972; Stivers and Enfield, 2010). The second research question asks what types of question forms children use when exploring a museum exhibit. We hypothesize that, like adults, children will use a variety of questions when interacting with their caregivers during museum exploration. We predict that, like adults, children will use more closed question types than open-ended (wh-) ones. As this type of child question analysis has not yet been conducted in this setting, this work is exploratory in nature.

Our final research question explores both the relationship between caregiver and child question usage and how question usage relates to language. Questions promote participation in a dialogue between individuals since they invite a response. During the turn-taking of an interaction, children are given an opportunity to practice language, learning from the more complex adult linguistic models and rules of conversational discourse (Bohannon and Bonvillian, 1997; de Rivera et al., 2005). Based on the same prediction above where we expect more closed questions than open-ended questions by both adults and children, we expect that there is a positive correlation between the amount of each of these types between caregivers and their children. In addition, we ask if the number of open-ended questions relates to a child’s language level with the hypothesis that these two measures will covary. As noted, open-ended questions provide the opportunity for a variety of types of responses with fewer constraints (Hargreaves, 1984; de Rivera et al., 2005). Across settings, these types of questions enhance cognitive abilities by supporting higher-order thinking, judgment, and reasoning skills (Hargreaves, 1984; Hoff-Ginsberg, 1986; Roth, 1996; Rowland et al., 2003; Valian and Casey, 2003). By encouraging a more elaborate response (beyond yes or no) and potentially a longer dialogue, there are language structure advantages with evidence showing that, in the case of school-age students, this increases vocabulary (Harlen, 1999). For preschool-aged children, de Rivera et al. (2005) showed that children are sensitive to question type and will produce more multiword utterances following educators’ open-ended and topic-continuing questions. Based on these previous findings, we predict that the number of open-ended questions will positively correlate with a child’s language level (when controlling for the age of the child).

The overall goal of the current study is to fill in gaps in the literature on the types of question forms caregivers use in a learning-based children’s museum environment with preschool-aged children. Child questions as well as child language level are examined in relationship to their caregiver’s question form use. This work complements learning research that examines the association between the quality of caregiver-child interactions and short- and long-term learning outcomes (Andre et al., 2017; Callanan et al., 2017; Sobel et al., 2022).

## 2 Materials and methods

### 2.1 Participants

Data were analyzed from 43 caregiver-child dyads. Thirty-eight caregivers self-identified as female/mother and five as male/father. All participants were the legal guardians of the participating children. Adult participants were on average 38 years old (*M* = 37.9 years; *SD* = 5.82 years; *range* = 22 to 48 years). Child participants ranged from 3 to 6 years old and were on average 4 years and 10 months old (*M* = 58.25 months; *SD* = 11.26 months; *range* = 39 to 81 months). All participants self-identified as white/Caucasian. Average family income was $91,063 (*SD* = $48,921; *range* = $20,000–$200,000). Inclusion criteria for the children were that they were between 3 and 6 years old, had no reported speech/language, genetic, developmental, or neurological disorders, were native speakers of United States English, had normal or corrected-to-normal vision and hearing, and were able to provide verbal assent. Data from an additional 18 dyads were excluded from the analysis due to poor sound quality of audio recorded interactions (9), refusal or inability to wear the headset microphone (4), or technical difficulties (5). Finally, due to these same reasons, some dyads completed one exhibit but not the other or only had audio for one of the participants (child or adult).

The study received institutional review board approval. Informed consent was obtained from the caregivers/legal guardians at the beginning of the study. Recruitment was through the Children’s Museum of New Hampshire (CMNH) website and newsletters, social media pages (e.g., Facebook), flyers posted in the community, and by word of mouth. Dyads received free admission to the museum and a $20 gift card at the completion of the study. All participants were recruited from the seacoast region of New Hampshire (NH). These data are taken from the larger interdisciplinary Advancing Children’s Museum Engagement (ACME) study (Thorson et al., 2020, 2022; Mannesto et al., 2021; Mueller et al., 2021).

### 2.2 Procedure and materials

**Figure 1:**
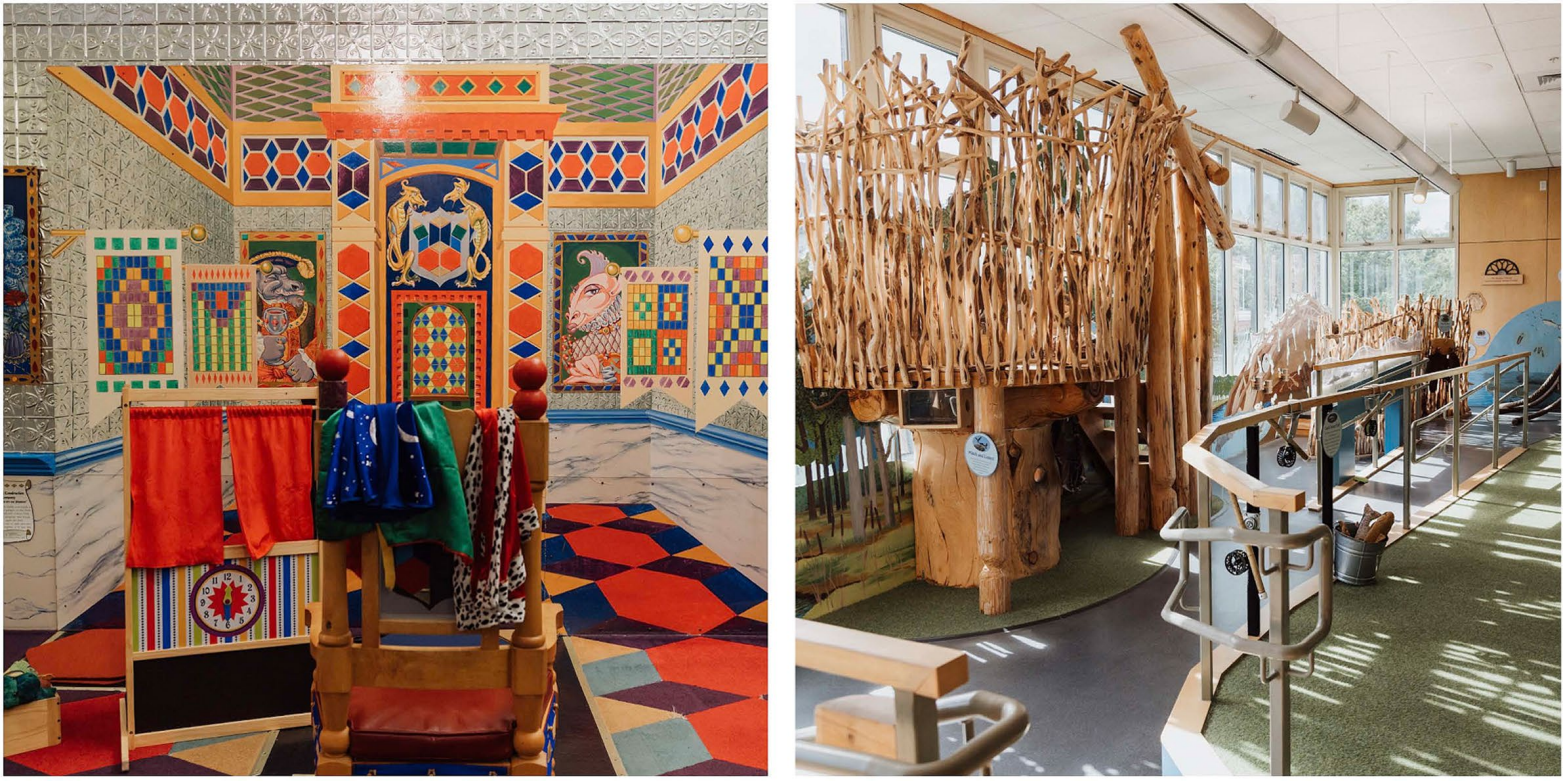
Images of the Pattern Palace (left) and Cocheco River (right) exhibits at the Children’s Museum of NH.

After obtaining caregiver consent and child assent, caregivers were administered a set of surveys as part of the ACME study. For the current study, only the demographic survey was used in the analyses. While the caregiver completed the surveys, the child was administered three subtests that make up the Core Language Score (CLS) from the Clinical Evaluation of Language Fundamentals Preschool Version 2 (CELF-P2; Wiig et al., 2004). The three subtests that make up the CLS are Sentence Structure (SS), Word Structure (WS), and Expressive Vocabulary (EV). After completing this initial phase, the dyads were invited to play in two different exhibits in the CMNH. The two settings were the Cocheco River Exhibit (referred to as River here) and the Pattern Palace (referred to as Palace here), see Figure 1 for an image of each exhibit. The order of exhibits was randomized and counterbalanced so that half of the participants started in the River exhibit and then went to the Palace, and the other half did the opposite. The materials in the River exhibit included informational learning materials aimed at facilitating conversation and play regarding animals, plants, and occupations related to NH river ecosystems. The Palace exhibit was approximately a third of the size of the River exhibit and included learning materials that focused on abstract shapes, various colors, and pattern recognition and creation. In addition to the standard materials available in each exhibit, a series of novel materials were provided exclusively for caregiver-child dyads during their time in the exhibit and were not available during the standard museum hours. Blocks, felt boards, castle figurines, cup and scroll patterning materials, and capes were provided in the Palace, and animal backpacks, raft building materials, an ecosystem matching game, and a magnetic fishing game in the River. These materials were selected with the guidance of the museum directors with the goal of stimulating creativity, interaction, and novel play. Items were donated to the museum upon completion of the study.

Dyads were provided a total of 20 minutes of play in each exhibit, 15 minutes of free play and 5 of clean-up. The caregivers were not made aware of the specific research interest of question usage. During this time, access to the exhibit was closed to the other museum patrons to allow less disruption during dyadic interaction. Sessions in each exhibit were audio recorded using Bluetooth Samson XPD1 audio headset microphones. The headset microphones linked to a laptop computer where the two headsets (adult and child) were assigned to the left and right recording channels using the Mac audio MIDI setup and then synced with Garage Band for recordings made at 48 kHz. After both recordings were complete from the two exhibits, the recordings were saved as WAV files for analysis.

### 2.3 Analysis and reliability

Data from all dyads were transcribed and annotated. Due to poor audio quality, refusal to wear the headset, and technical difficulties, additional data were removed per exhibit or participant; this is described in more detail in the Results section. Language from each session was transcribed and analyzed using the Systematic Analysis of Language Transcription (SALT) software (Miller and Iglesias, 2012). All questions were extracted from the full WAV files, saved as individual WAV files, and annotated using the acoustic software Praat (Boersma and Weenink, 2018). Using a Praat script, each file was opened and a corresponding TextGrid was created, annotated, and saved. Each TextGrid consisted of five tiers: Vowel, Syllable, Word, Utterance, Question Type, and Comments. For this study, the output from the Question Type and Comments tiers were utilized. A set of question categories was created based on past literature and annotated in the Question Type tier (Leach, 1972; Parker and Pickeral, 1985; Gunlogson, 2002; Iwata, 2003; Stivers, 2010). See Table 1 for a list of question categories, definitions, and examples. The Comments tier in Praat was used to note anything unique or inconsistent about a sound file (e.g., unintelligible portions, whisper).

**Table 1:** Definitions and examples of each question type annotated for the child and caregiver (adult) data.

**Question type**	**Definition**	**Caregiver examples**	**Child examples**
**Polar** (yes-no questions)	Elicits a response of *yes* or *no* from the questionee.		
*Polar Interrogative* *(inverted aux)*	A polar question that is syntactically constructed as an interrogative typically with auxiliary verb inversion (i.e., aux-inversion).	*Did it fall off again?* [R] *Oh boy, are we goin’ overboard?* [R] *Do you know what a knight does?* [P]	*Do you wanna be a stingray?* [R] *Is that what noise they make?* [R] *Is that the king?* [P]
*Polar Declarative (non-inverted)*	Differ from interrogative questions in that they assume the questioner already has information about the situation (Gunlogson, 2002). Syntactically they are constructed as declaratives without aux-inversion. Use prosody to indicate question status.	*You’re gonna do that one?* [R] *You’re gonna go get those off the window?* [P]	*Sticks that beavers would use?* [R] *That’s where the magnet is?* [R] *This is a king’s castle?* [P]
*Polar Tag*	Express “maximum conduciveness,” meaning they coerce particular answers to questions (Stivers, 2010).	*Yeah, it builds like a dam, right?* [R] *Yah, feeding a dragon is more interesting, right?* [P]	*How bout I will put them in the net and you can do it, okay?* [R] *Let us play with something else, kay?* [P]
**Alternative** (either/or questions)	Usually involve the questionee needing to choose between two options. They can involve two separate questions or clauses conjoined by *either*/*or* (Stivers, 2010).	*Can mommy go first or do you wanna go first?* [R] *Shall I be the knight or the dragon?* [P]	[None in River] *Your shoes or my shoes?* [only one in Palace]
**Echo** (repetition questions)	Function as a way for the questioner to gain clarification or express certain emotions, like surprise or disbelief (Iwata, 2003).		
*Echo Repetition*	Occurs when the questioner repeats a part or entirety of the preceding utterance.	*We’re going swimming?* [R] *You wanna be a dragon?* [P]	*Woodpeckers?* [R] *A crown?* [P]
*Echo Wh-word*	Occurs when a wh-word replaces part of the preceding utterance to form a question. Similar to *in-situ* wh-questions.	*The legendary what?* [R] *It’s a what?* [P]	*[He] is doing what?* [R] [None in Palace]
**Content** (wh-questions)	Include the words w*ho, what, when, were, why,* and *how*, which typically come at the beginning of the question (Stivers, 2010).	*What do you think will happen?* [R] *What is your first rule as queen?* [P]	*What is this supposed to do?* [R] *What can we make?* [P]

[P] denotes examples from the Palace exhibit and [R] from the River exhibit.

From Praat, the outcome variable of question type was analyzed for adults and children by exhibit. From SALT, adult and child language measures were extracted that capture linguistic complexity and diversity: total number of utterances for adults and children, children’s mean length of utterance in morphemes (MLUm), children’s total number of words (NTW), children’s number of different words (NDW), and children’s moving average type-token ratio (MA_TTR; Moving-Average TTR; calculated by dividing moving-average NDW by moving-average NTW). These measures reflect linguistic aspects including general quantity/amount of language (i.e., total number of utterances, NTW), morphosyntactic complexity (MLUm), and lexical complexity (i.e., NDW, MA_TTR). Overall child language ability was also assessed using the standard CLS from the CELF-P2.

For the question-type coding, one rater classified each utterance as belonging to one of the seven question form categories based on the definitions in Table 1. These categories were based on work from syntax and semantics defining schemas and sub-types of questions (Leach, 1972; Gunlogson, 2002; Iwata, 2003; Stivers, 2010; Stivers and Enfield, 2010; Sadock, 2012). Additionally, see the Supplementary material for a *post hoc* exploratory analysis breaking down the Content (wh-) question category further by looking at the percentage of wh-word sub-types (i.e., ‘what,’ ‘why,’ ‘where,’ ‘how’) produced by the adult caregivers and children across the two exhibits. This exploratory analysis revealed that both groups used ‘what’ questions most frequently. For interrater reliability, 15% of the data was classified by a second rater. Percentage of agreement was calculated by adding up the number of utterances that were in agreement between two independent raters and dividing that number by the total number of utterances that the two raters produced. This revealed that the percentage of agreement was 90%, indicating a high level of agreement. Additionally, the interrater agreement between two raters was assessed using Cohen’s kappa statistic. The calculated kappa coefficient was *κ* = 0.87 (95% CI [0.82, 0.91]), indicating almost perfect agreement among the raters.

There were several steps taken to ensure accurate SALT transcriptions. First, comprehensive trainings and practice transcriptions were completed by all transcribers and compared to a gold standard. Second, once a transcriber completed a transcript, a second transcriber verified all transcripts to ensure accurate representations. Disagreements were noted, discussed, and resolved with the original transcriber until a consensus was achieved. Finally, a randomly selected 10% of the transcripts from the two exhibits were transcribed/coded by a second transcriber. Interrater reliability for the SALT transcriptions was calculated using intraclass correlation coefficients (ICC) for the measurements of interest for the children and adults. ICC estimates and their 95% confidence intervals were calculated using SPSS 29 (IBM Corp., 2024) based on average measures, absolute agreement, 2-way mixed-effects model. For all measurements, an ICC (2,1) of 0.92 or higher was observed, suggesting excellent agreement among the raters: total number of utterances-adults: 0.98 (95% CI[0.96,0.99]); total number of utterances-children: 0.99 (95% CI[0.97,0.99]); MLUm-children: 0.92 (95% CI[0.64,0.97]); NTW-children: 0.97 (95% CI[0.98,0.99]); NDW-children: 0.98 (95% CI[0.98,0.99]); MA_TTR-children: 0.93 (95% CI[0.73,0.98]).

#### 2.3.1 Statistical analysis

Data analysis was completed in R (R Core Team, 2023) using the following packages: *dplyr* (Wickham et al., 2023), *tidyverse* (Wickham et al., 2019), *ggplot2* (v3.3.3; Wickham, 2023), and *viridis* (color-blind palette; Garnier et al., 2023). Statistical analyses were conducted using generalized linear mixed models (GLMMs) to assess significance. All GLMM analyses were conducted in R with the following packages: *coda* (Plummer et al., 2006), *lme4* (Bates et al., 2015), *MCMCglmm* (Hadfield, 2010a), and *parallel*. For analyses with a binary categorical response variable, GLMMs were conducted with Condition (Palace; River) and Group (Adult; Child) as fixed factors and a random effects structure nesting participant ID within dyad ID. A multinomial mixed-effects logistic regression was selected when the response variable consisted of more than two categories. In this case, a reference category is required to which each of the other categories are compared. Fixed and random effects were modeled in all GLMM models; random intercept and slope for participants and a random intercept for participants nested within the Group variable were estimated. Following Barr et al. (2013), this was the maximal random effects structure justified by the study design. Participant ID was used for the random effect, which utilized a unique identifier for each participant and where the model naturally nests participant in each dyad. The fixed effects were Group and Condition for the MCMC GLMM model as well. The intercept was suppressed in the models as recommended by Hadfield (2010a).

Furthermore, a Bayesian framework utilizing Markov chain Monte Carlo (MCMC) methods to implement model fit was selected given model complexity, the ability to obtain unbiased parameter estimates for categorical, non-normal dependent variables with more than two levels (particularly when data are unbalanced), and its suitability to interpret and assess significance of the parameters (Hadfield, 2010b). This type of model requires specification of prior probabilities and yields estimates of posterior probabilities. A burn-in period of 10,000 with 85,000 iterations and a thinning interval of 25 were used for a sample size of 3,000. The prior structure was based on recommendations of Hadfield (2010b, p.23) for categorical mixed-effects models and was non-informative. Fixed priors were set at 0.5 for all diagonal terms and 0.25 for all off-diagonal terms (covariance). Model convergence of four independent Markov chains was checked using the Gelman-Rubin’s criteria (Gelman and Rubin, 1996), which produces a potential scale reduction factor (PSRF) where a conservative *R* factor value of <1.1 is the criterion for convergence. The *R* factor for each of the fixed effects for our model was always between 1.0 and 1.06, indicating good mixing of the chains. To evaluate the relevance of the fixed effects in this framework (and which is more in line with a Bayesian analysis), model output of the posterior probability estimates includes a posterior mean given in log-odds (similar to a *β* estimate) and 95% credible intervals (similar to standard error). Additionally, the MCMC computation derives *pMCMC*, which are analogous to a *p*-value (more in line with the standard frequentists analyses). Both measures are provided by the MCMCglmm function. See Supplementary material for details on the statistical models including the R code.

Finally, Spearman rank correlations were used to explore the relationships between question types and child language ability with follow up partial Spearman rank correlations conducted to control for differences in child age. A *post hoc* linear regression was run with the predictor variable of child age group (four groups: 3-, 4-, 5-, and 6-year-olds) and the outcome variable of proportion of open-ended to closed questions uttered by the adults. Due to data collection being halted due to COVID-19, the child age groups were unbalanced, and this led to difficulties with this analysis. Nonetheless, the assumptions for the linear regression were met and main findings are presented with additional details provided in the Supplementary material.

## 3 Results

A total of 3,661 questions were identified in the adult data (Palace: 1790; River: 1871) and 1,164 in the child data (Palace: 567; River: 597). From these, a total of 255 utterances were excluded due to intelligibility issues or poor audio quality (Adult: Palace: 22, River: 85; Child: Palace: 89, River: 59). A final total of 4,570 utterances were included in the analysis. See Table 2 for a breakdown of how many of each question type were identified by participant group.

**Table 2:** Utterance counts for each question type category by participant group along with grand totals.

Question type	Caregiver			Child			**Grand total**
	Palace	River	Total	Palace	River	Total	
Polar-Interrogative	579	524	1,103	123	142	265	1,368
Polar-Declarative	353	385	738	60	65	125	863
Polar-Tag	72	102	174	33	24	57	231
Echo-Repetition	135	199	334	15	7	22	356
Echo-Wh	5	13	18	0	1	1	19
Alternative	55	30	85	1	0	1	86
Content	569	533	1,102	246	299	545	1,647
**Grand total**	**1768**	**1786**	**3,554**	**478**	**538**	**1,016**	**4,570**

**Figure 2:**
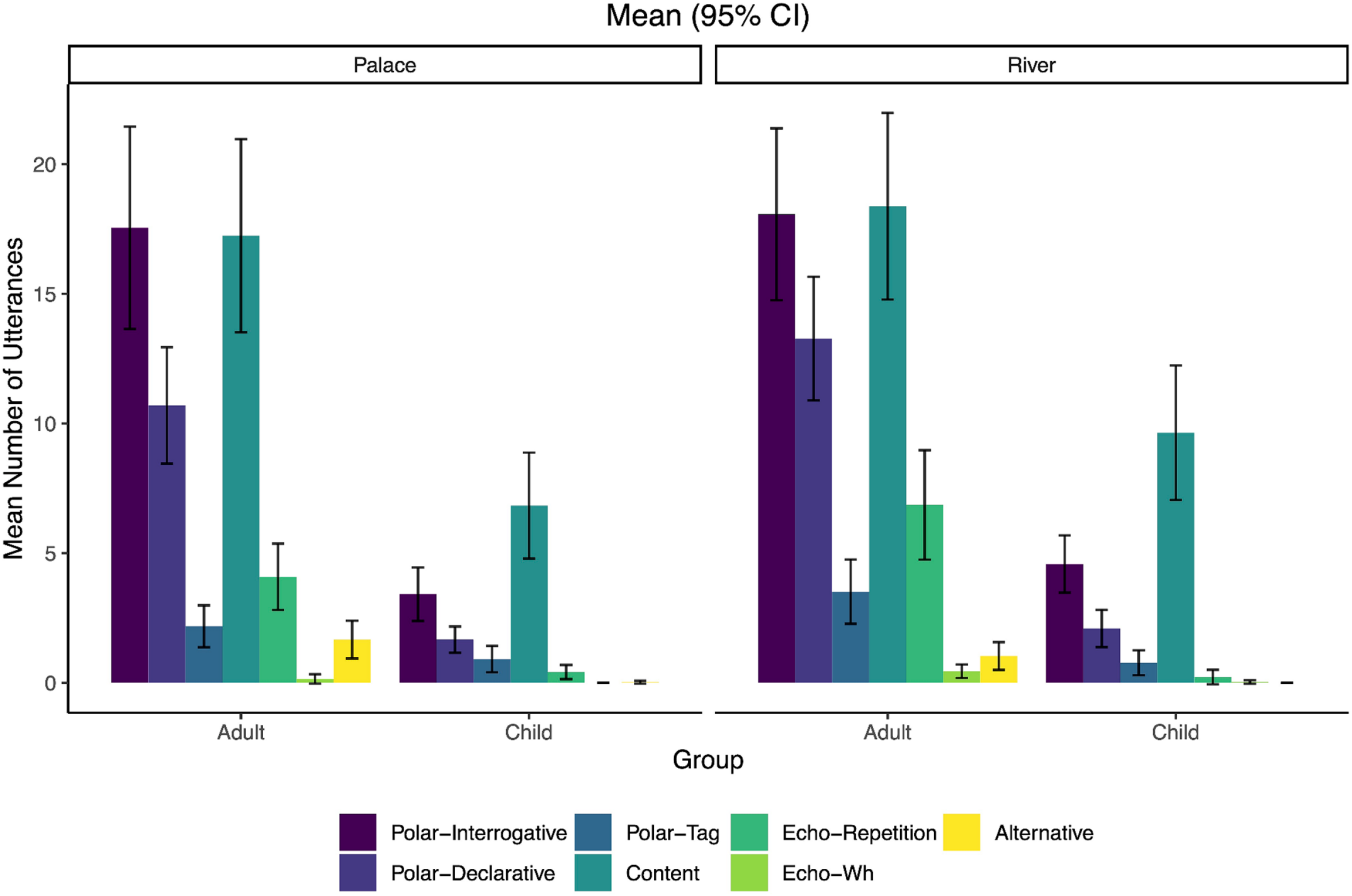
Mean number of utterances for all question category types by condition (Palace and River) and group (adults/caregivers and children). Error bars represent 95% confidence intervals.

**Figure 3:**
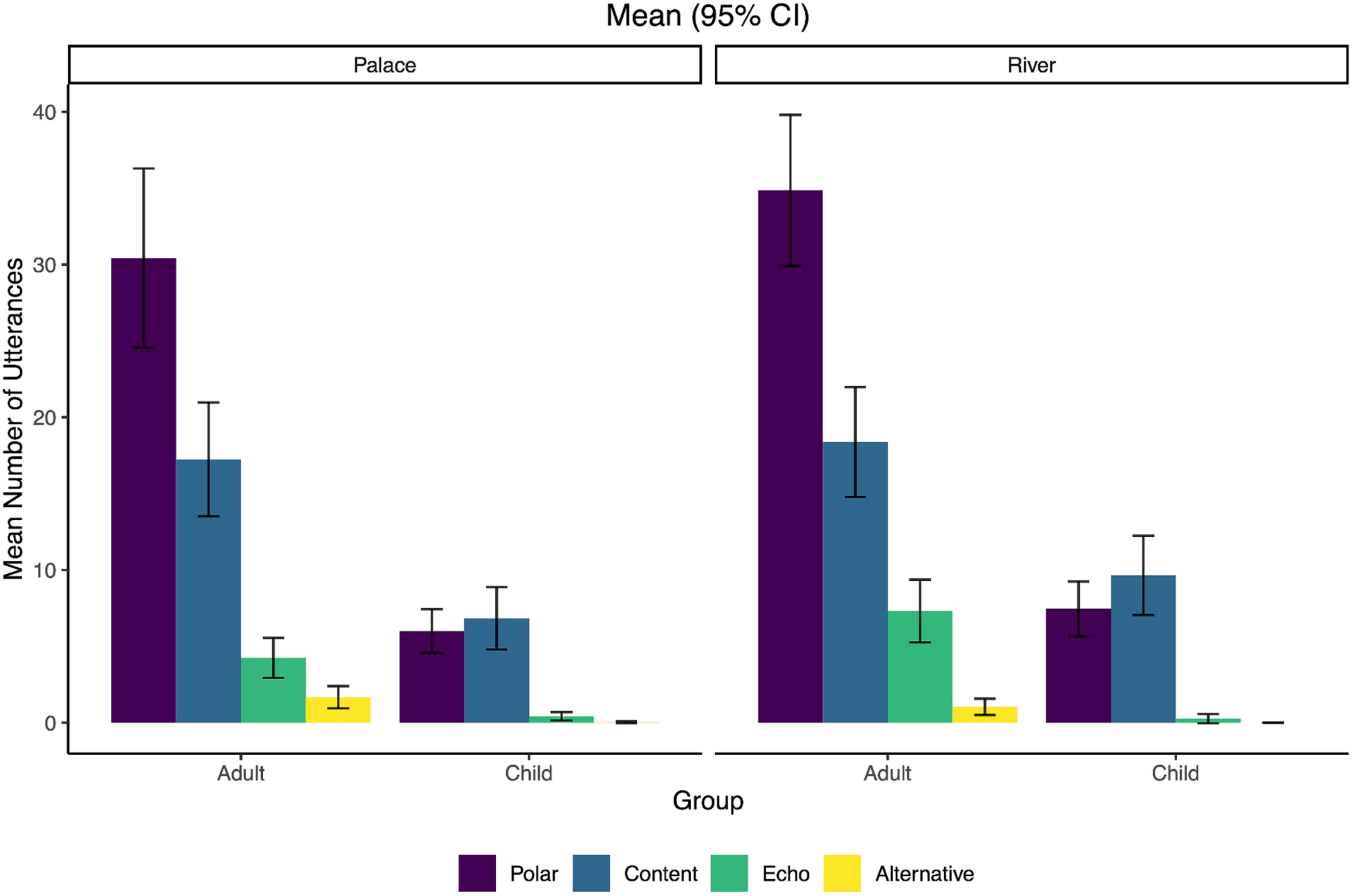
Mean number of utterances for each question sub-category type (polar, content, echo, and alternative) by condition (Palace and River) and group (adults/caregivers and children). Error bars represent 95% confidence intervals.

**Figure 4:**
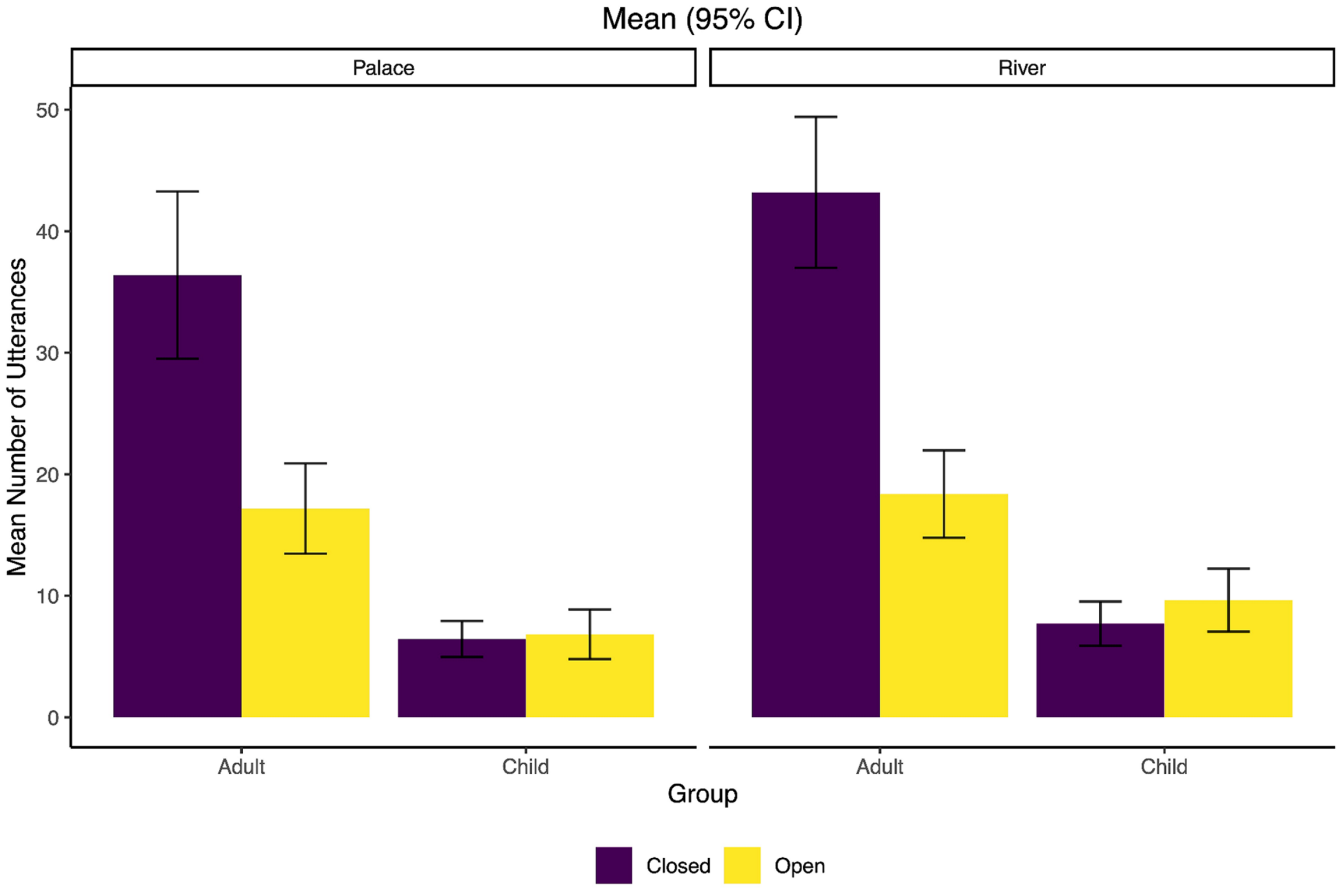
Mean number of utterances for closed vs. open question type by condition (Palace and River) and group (adults/caregivers and children). Error bars represent 95% confidence intervals.

Next, the mean of each question sub-category was analyzed as well as broader question categories. Figure 2 shows the means for all sub-categories while Figure 3 collapses the three polar question sub-types (interrogative, declarative, tag) into one broad ‘polar question’ category and the two echo question sub-types (repetition and wh) into one ‘echo question’ category. Finally, Figure 4 shows the means when the types are collapsed into the broadest categories of open-ended (content or wh-) versus closed (polar, echo, alternative).

We fitted a GLMM (estimated using ML and BOBYQA optimizer) to predict question type (closed versus open) with Condition and Group as fixed effects, along with a random effect of by-participant nested within dyad (see Figure 3). The model’s total explanatory power was weak (conditional *R^2^* = 0.11), and the part related to the fixed effects alone (marginal *R^2^*) equal to 0.05. Model output is summarized in Table 3. There was a significant effect of Group, but not of Condition, indicating that there are differences between open-ended and closed question use between children and adults, but no significant differences between the River and Palace conditions. The significant interaction of Group by Condition indicates that the differences in Group are mediated by Condition, with a larger difference between open-ended and closed question types for the adults in the River condition compared to the Palace condition was found.

**Table 3:** GLMM model output for the categorical dependent variable of broad question type (open vs. closed) with fixed factors Condition (River vs. Palace) and Group (adult/caregiver vs. child).

**Parameter**	***β***	***SE***	***Z***	***p***
(Intercept)	−0.82	0.10	−8.51	< 0.001
Condition = River	−0.08	0.08	−0.93	= 0.353
Group = Adult	0.80	0.16	5.01	< 0.001
Condition = River*Group = Adult	0.39	0.17	2.30	= 0.022

Next, a multinomial logistic regression using *MCMCglmm* was conducted to determine how the variables of Condition and Group affected the distributions across the three sub-types of questions (polar, content, and echo; see Figure 3). The alternative question type was omitted from the analysis due to too few samples across the two groups. The reference group for the response variable was set to polar questions, while adult was the baseline condition for the fixed effect of Group and Palace was the baseline condition for the fixed effect of Condition. Random effects were also included consisting of intercept and slopes for by-participant (dyad) effects. Results of the analysis are summarized in Table 4. After statistically controlling for random effects, the content and echo questions were predicted to be significantly less common than the polar questions (reference category) at baseline (adult for the Palace condition), as shown by the significant negative mean logit values. This is shown by content and echo question intercept log-odds estimates differing significantly from zero. The model showed that when adults were in the River condition, there was a significant increase in the likelihood of producing echo questions relative to polar questions, compared with the reference likelihood (rate of content- and echo-to-polar responses in the Palace condition). These findings suggest that the increase in polar questions in the Palace condition compared to the River condition is due to the lower prevalence of polar questions and more echo questions in the River condition when compared to their rates in the Palace condition.

**Table 4:** GLMM for the multinomial distribution of polar, content, and echo question response types; polar was the reference level.

**Factor** (comparison to baseline for adult in palace condition)	**Response type**	**Posterior mean** **(log-odds)**	**95% CI (log-odds)**	***pMCMC***
**Fixed effects**				
(Intercept)	Content	−0.75	[−0.99, −0.51]	<0.001
(Intercept)	Echo	−2.41	[−2.76, −2.06]	<0.001
Condition = River	Content	−0.02	[−0.21, 0.18]	=0.836
Condition = River	Echo	0.55	[0.23, 0.84]	<0.001
Group = Child	Content	0.79	[0.29, 1.22]	=0.003
Group = Child	Echo	−0.83	[−1.63, −0.07]	=0.029^*^
Condition = River:Group = Child	Content	0.45	[0.06, 0.89]	=0.029
Condition = River:Group = Child	Echo	−1.08	[−2.17, −0.04]	=0.029
**Deviance** (DIC)				7699.96

Fixed factors are Condition (Palace vs. River) and Group (adults/caregiver vs. child), with Palace and child set as the baseline levels of Condition and Group. Type I error rates are pMCMC values (Baayen et al., 2008) with ^*^p < 0.05, ^**^p < 0.01, and ^***^p < 0.001; pMCMC, probability that the parameter estimate includes zero.

Group (adult vs. child) significantly affected the relative rates of the variant question types. For children, there was a significant difference (i.e., more) in the relative rate of content questions in the Palace condition compared to the reference rate (i.e., rate of content to polar questions for children in the River condition), and a significant decrease in relative rate of echo questions in the Palace condition as compared to the reference rate. Finally, there were significant interactions between Group (adult vs. child) and Condition (Palace vs. River) on relative rates of both question types (content and echo) compared with rates in reference conditions. That is, being in the Palace condition significantly related to the variant rate distributions compared with the River condition reference rates of questions for the children. A significant interaction between the Condition and Group for the question variants implies that the rates of content and echo questions in the Palace condition were distinct from the rates in the River condition. For the children, there is a significant increase of content questions relative to polar questions in the River condition as compared to the Palace. There is also a significant decrease in echo questions relative to polar questions in the Palace condition as compared to the adults in the River condition. Overall, this analysis shows that both adults and children have higher rates of polar questions in relation to content and echo questions and that while the condition did not have a direct impact on question usage, it does show that it mediates the rates of some of the variants in relation to the polar questions.

To explore the effect of child age on caregiver question use, a *post hoc* linear regression model was implemented to predict the amount of open-ended to closed questions used by the adult caregivers based on the age of the children (separated into four age groups: 3-, 4-, 5-, 6-year-olds). The model showed that the child age groups did not significantly predict the proportion of open-ended questions produced by the caregivers [*F*(1,20) = 1.24, *p* = 0.279, *R^2^* = 0.06], though visual inspection reveals that there was a small increasing trend in the use of open-ended questions by caregivers of 3- to 5-year-olds (change of ~0.06). See Supplementary material for full model summary results and a figure depicting mean values and standard errors of proportion of open-ended to closed questions for caregivers by child age group.

Spearman rank correlations were computed to assess relationships between language and question type outcome measures. See the Supplementary material for a full correlation matrix between all variables, only the significant correlations will be reported here. Potential associations between the child language measures (total utterances, MLUm, NDW, NTW, MA_TTR, and CELF) and the child question measures (number of total questions, open-ended, and closed) with the adult measures (total utterances, and number of total questions, open-ended, and closed) were explored. There were no significant correlations between the child question measures and any of the adult measures or child language measures (see Supplementary material). Significant correlations were found for the child total number of utterances, child CELF scores, child MLUm, and the child MA_TTR measures with the total number of adult utterances and the total number of adult questions (total, open-ended, and closed) (Supplementary material). Additionally, the adult measures were all highly correlated with one another, with the total number of adult utterances significantly and positively correlating with the total number of adult questions (*r*[19] = 0.78, *p* < 0.001), number of adult closed questions (*r*[19] = 0.79, *p* < 0.001), and number of adult open-ended questions (*r*[19] = 0.58, *p* = 0.006).

For the significant correlations involving child measures, follow-up semi-partial Spearman correlations were computed to take into account the effect of child age. While controlling for the age of the child, there were significant positive correlations between the total number of child utterances and the number of adult utterances (*r*[19] = 0.64, *p* = 0.003), the total number of adult questions (*r*[19] = 0.50, *p* = 0.026), and number of adult closed questions (*r*[19] = 0.48, *p* = 0.033).

Additionally, while partialing out the effect of child age, there were negative correlations between the total CELF score of the child and the total number of adult utterances (*r*[19] = −0.48, *p* = 0.031), total number of adult questions (*r*[19] = −0.61, *p* = 0.004), number of adult closed questions (*r*[19] = −0.57, *p* = 0.008), and marginal significance for number of adult open questions (*r*[19] = −0.44, *p* = 0.055). Finally, when controlling for child age, the child MA_TTR was significantly negatively associated only with the total number or adult utterances (*r*[19] = −0.57, *p* = 0.0049), but not with the total number of adult questions (*r*[19] = −0.34, *p* = 0.139) or the number of adult closed questions (*r*[19] = −0.26, *p* = 0.262). None of the follow-up semi-partial Spearman correlations for child MLUm were significant with the adult measures when controlling for child age.

## 4 Discussion

The primary aim of this study was to analyze the different question forms used by caregiver-child dyads while playing in a children’s museum. Our first and second research questions focused on what specific types of question forms adults and children use during this interaction. The findings show that adults used more closed (~68%) than open-ended (wh-) questions (~32%), which aligns with previous research showing the greater use of closed question forms among caregivers and teachers of young children (Newport et al., 1977; Wittmer and Honig, 1991; O’Brien and Bi, 1995; de Rivera et al., 2005; Bay and Hartman, 2015). Specifically, adults utilized more polar (yes-no) than content (wh-) questions, which also follows previous work showing that yes-no questions are the most frequently occurring question form in similar learning environments (Newport et al., 1977; de Rivera et al., 2005). The data also revealed that of the polar question types, the polar interrogatives (with aux-inversion) were the most frequent for adults, with polar declaratives (without aux-inversion) being the next most frequent, and finally very few polar tag questions used. The children showed a different overall pattern than the adults and used comparable amounts of open-ended (~50%) and closed question forms (~50%), with polar and content questions largely making up these categories, respectively. Of the polar questions produced by children, they roughly reflected the adult pattern and produced mostly polar interrogatives (with aux-inversion), then polar declaratives (without aux-inversion), and then very few polar tags. This also aligns with previous research showing that children tend to produce more yes-no questions than wh-questions (Rowland, 2007). Neither participant group had very many occurrences of either type of echo question or the alternative question-type, though adults used slightly more of both types than the children.

When comparing question forms across the two museum exhibits, there were no significant differences in the ratios of open-ended (wh-) to closed questions based on whether the dyad was engaging in play in the River or the Palace. This reflects the finding that both groups were consistent in their overall question usage across the two exhibits and that the setting did not impact the ratio of open-ended to closed questions. Being in one exhibit or the other did not create a meaningful difference in the use of these two broad question categories, but the pattern of results varied between the children and the adults. This can be observed via the nearly equal use of open-ended and closed question types for the children, but more closed than open-ended forms among adults. Additionally, the adults used a larger quantity of closed questions in the River than in the Palace exhibit, creating a slightly greater difference between closed and open-ended types for that exhibit.

Additionally, a *post hoc* analysis was conducted to examine the potential impact of child age on the proportion of open-ended questions used by caregivers. Based on literature showing that parents adapt their communication to children to support them while also challenging them (e.g., Vygotsky’s Zone of Proximal Development), it might be expected that there would be an increase in the use of open-ended questions over development by caregivers (Vygotsky, 1978; Bruner, 1983; Rowe, 2012). In contrast, other research has shown a decrease in the amount of open-ended questions during the preschool years used by mothers during narrative tasks (Kuchirko et al., 2016). Here, the data show that while there is a small increase in the amount of open-ended (wh-) questions asked to children as their age increased from 3 to 6 years old, this change was not significant. Due to the unbalanced sample sizes in the child groups, this analysis was underpowered making it difficult to draw any firm conclusions. Future work would benefit from additional investigations into how child age affects the types of questions that caregivers utilize during museum exploration.

For the adults and children, the analysis of question type use between polar (interrogative, declarative, tag), content (wh), and echo questions (repetition, wh) showed that the polar questions were significantly more frequent than the content and echo types (alternative questions were not part of this analysis due to their low occurrence rates). As previously discussed, this higher frequency of polar yes-no questions aligns with the literature (e.g., Newport et al., 1977; Rowland, 2007). Our data adds to this body of work by demonstrating that these ratios of occurrence are also held by caregivers during play in a children’s museum learning environment. The findings also highlight the use of a range of specific question forms by both caregivers and children (e.g., polar, content, echo). When considering the specific exhibits, adults used more polar questions in the Palace than in the River exhibit, and relatively more echo questions in the River than in the Palace. This could potentially be due to the environment and materials of each exhibit. The Palace is a smaller space that is focused on learning concepts around patterns, shapes, and colors. By comparison, the River is a much larger space with learning materials focused on facilitating conversation about animals and plants. Thus, the exhibits themselves may be driving some of these differences. Follow-up work is necessary to tease apart the specifics of how varying elements of museum exhibits (e.g., focus, space, materials, directions) may affect language use during these types of dyadic interactions.

Furthermore, adults and children showed different patterns in their use of polar, content, and echo questions depending on the exhibit. Children used more content questions in the Palace than in the River, reflecting the adult pattern. Children also used relatively fewer echo questions than the adults, but, like the adults, they used more echo questions in the River than in the Palace. Echo questions may be used for confirmation or clarification in an interaction, to close a gap in information, or to express surprise or incredulity (Moulton, 1987; Takahashi, 1990). Thus, one possibility is that adults may seek this type of verification in engagement from the children more often than children do from their caregivers. In general, both groups used more polar questions than content or echo ones across the Palace and River exhibits with the specific exhibit having a small role in mediating the rate of use.

Our third research question sought to examine potential relationships between the question forms utilized and the language measures analyzed. Within the adult measures, there were medium to strong positive correlations between the number of open-ended questions, closed questions, the total number of questions, and the total number of utterances, showing that adults who had more utterances also asked more questions and adults who asked more questions also used more open-ended and closed questions (with the correlation to closed questions being very strong). In addition, the more that an adult uttered and asked questions, the more utterances the child produced as well, even when controlling for the age of the child. In particular, there was a positive association between the number of closed questions an adult asked and the number of utterances the child produced. Without analyzing the makeup of the child responses, it is evident that more question asking by the caregiver led to more child utterances during play. This is in line with work showing that both question types (open-ended and closed) successfully encourage child responses (Honig and Wittmer, 1982; Wittmer and Honig, 1991).

In terms of child language, significant correlations were found for three child measures: morphosyntactic complexity (measured via MLUm), lexical diversity (measured via the moving-average type-token ratio, MA_TTR), and general language ability (measured via the child’s standard Core Language Score (CLS) on the CELF-P2). The morphosyntactic complexity and language ability measures both negatively correlated with adult question types (open-ended and closed) as well as the total number of questions and utterances of the adults. The lexical diversity measure negatively correlated with the total number or adult utterances, indicating that the more the adult spoke, the less lexical diversity was present in the child’s language. When the age of the child was controlled for, the overall child language ability measure was still negatively associated with the total number of adult closed questions, adult open-ended questions, and the adult total number of questions. At first consideration, it may seem counterintuitive that the more questions an adult asks, the shorter the child utterances and lower their language score. However, this follows previous work showing that children respond with less linguistically complex utterances to closed questions versus open-ended ones (Harlen, 1999; Çakır, 2016; Rowe et al., 2017). Polar closed-ended questions only require a limited word response, which will then impact the overall mean length of the child’s utterances. Notably, the negative association between higher question usage by adults held for the child’s language scores. This potentially means that these caregivers may use closed questions more frequently across environments, not only during play in a children’s museum. Past literature has supported the use of open-ended wh-questions to stimulate expanded responses and build vocabulary in learning environments (Wood and Wood, 1983; Harlen, 1999). The connection between lower child lexical diversity and higher amount of caregiver language potentially reflects how caregivers often model or expand their language for children with lower expressive vocabulary (Hart and Risley, 1995). Future steps include analyzing how children respond to different types of questions, examining utterance length and complexity in response to different adult question forms and functions.

There are several limitations of this work regarding the study approach. First, this study did not directly analyze the types of child responses to the different question forms. As this is the first in-depth analysis of caregiver question forms with this aged population in a children’s museum setting, the primary goal of the work was broader in nature. Future continuations of this project plan to examine child responses more closely and how they may differ (or not) in response to caregiver question forms. In line with this limitation, this study focused on question *form* and not *function*. To establish a baseline of the types of questions that caregivers were using in this setting, question form was the focus of this initial investigation. It is well established that the function of a question stimulates different types of engagement and responses (De Ruiter, 2012b). For example, topic-continuing or topic-initiating questions impact conversation and child language in unique ways, with topic-continuing supporting more complex linguistic responses from children (de Rivera et al., 2005). Clearly defining sub-types of questions based on both *form* and *function* in conjunction would offer additional insight into their impact on child language use in a museum setting. For example, a more nuanced coding schema could differentiate broad versus narrow focus wh-questions, which may lead to a different type of division for open-ended versus closed questions. Future extensions of this project plan to quantify questions along functional dimensions to analyze how caregiver language influences concurrent child language abilities.

Relatedly, the current work did not specify between an initiating question versus a follow up question; this would be another aspect to differentiate when analyzing questions using a more functional approach (see Fitneva, 2012 and Ronfard et al., 2018 for reviews on the epistemic and social nature of questions and the development of question-asking during early childhood, respectively). This difference may impact the amount of yes-no questions asked by the caregiver if their child does not first reply to a bid with more open types of questions. Additionally, continuing to analyze the data from multiple perspectives aids in fully understanding question asking by children across research areas (see Ronfard et al., 2018 for a review of child questions across disciplines). For example, future work may consider examining behavioral aspects of parenting, given research showing that it may play a role in explaining associations between caregiver verbal abilities and that of their children (Prime et al., 2020). Another consideration for future work is to include visual in addition to audio information during the analysis in order to capture gesture-based information related to question-asking (e.g., eye gaze, head-nod), which has been shown to influence the function and interpretation of a question (De Ruiter, 2012b).

Another limitation of this study is the composition of the sample. Though there was a high number of questions analyzed, the final sample size was smaller than the initial target due to the impact of COVID-19—data collection was forced to terminate early given local lockdown measures. This created unequal sample sizes for each child age group (from 3- to 6-years-old). Additionally, there was not a large range in the socioeconomic status (SES) of the families. SES can be calculated in a variety of ways with most metrics relying on family income and/or parental education levels to define the category (Duncan and Magnuson, 2003; Vasilyeva et al., 2008; Hackman and Farah, 2009). The impact that SES has on adult and child language has been well documented (e.g., Hart and Risley, 1995; Rowe and Snow, 2020). For this study, there was a very limited sample in terms of SES stratification with only nine dyads classified as lower SES based on parental education levels (Mueller et al., 2021). Due to the limited ability to detect differences with a smaller sample, we were not able to incorporate the role of SES into our current research questions. Nevertheless, this is an important and open area for future research, particularly when considering caregiver language during children’s museum interactions. Finally, it is important to note that our findings are limited to United States English speaking-individuals in the seacoast region of New Hampshire. We would not necessarily extend these results to cultures where children asking questions to adults (or vice versa) have the same set of expectations (Hoff and Tian, 2005). Furthermore, the population was also racially and ethnically homogenous with all participants self-reporting to be white/Caucasian. Limitations of the current study highlight the need for continued research as well as specific areas for future investigation including additional analyses and the inclusion of a more diverse sample population.

It is important to note that this work would not be possible without a successful partnership with the Children’s Museum of New Hampshire. Recent literature reviews discuss the value of university researchers working with museum directors and practitioners to better understand child learning and aspects related to cognitive development in these settings (Callanan, 2012; Sobel and Jipson, 2015; Callanan et al., 2020). It is through shared goals and a mutual professional relationship that these groups can come together to be able to aid visitor and child engagement and learning. Community engagement is central to this type of engaged scholarship and our work aims to foster this mutually beneficial collaboration.

In conclusion, how caregivers and children interact in a museum setting is an ongoing area of research. This study adds to the literature by contributing analyses from younger children (3- to 6-year-olds) who are playing with their caregivers in a children’s museum. Our focus was to examine the question forms of both adults and children during natural play and identify potential associations with child language abilities. We found that adults largely used closed questions, specifically polar interrogatives, and that children were much more balanced in their use of closed and open-ended (wh-) question forms. Children of parents who asked more closed questions also exhibited shorter utterance lengths and lower overall language abilities. These findings contribute to our understanding of how the use of unique question forms impacts language levels and dyadic interactions with the need for continued research on the function of these dynamic question exchanges.

## Data availability statement

The datasets presented in this article are not readily available because the deidentified datasets analyzed for this study are only available upon request from the authors pending institutional review approval. Requests to access the datasets should be directed to JCT, jill.thorson@unh.edu.

## Ethics statement

The studies involving humans were approved by University of New Hampshire-Durham Institutional Review Board for the Protection of Human Subjects in Research (IRB #: Legacy-UNH-7022). The studies were conducted in accordance with the local legislation and institutional requirements. Written informed consent for participation in this study was provided by the participants’ legal guardians/next of kin.

## Author contributions

JCT: Conceptualization, Data curation, Formal analysis, Funding acquisition, Investigation, Methodology, Project administration, Resources, Software, Supervision, Validation, Visualization, Writing – original draft, Writing – review & editing. JMT: Data curation, Investigation, Project administration, Resources, Writing – review & editing. KN: Funding acquisition, Resources, Writing – review & editing.
