# Supplementary material for: Caregiver and child question types during a museum interaction
Source: Front Psychol. 2024 Jul 9;15:1401772. doi: 10.3389/fpsyg.2024.1401772 (PMC11263923; doi:10.3389/fpsyg.2024.1401772)
Supplement: Supplementary file 1 [file Data_Sheet_1.pdf]

Supplementary Material

1 Details of statistical modeling

This first part of the supplemental material gives details of statistical modeling for results presented in Tables 3 and 4.

1.1 Modeling for Results in Table 3

```
load.packages("lme4")
library(lme4)
```

This following was the full and best model after using the `drop1` command in R.

```
model.table3 = glmer(QuestionType4 ~ Condition*Group +
      (1 |DyadID:PartID),
      data = df_QT4,
      family="binomial")
```

1.2 Modeling for Results in Table 4

```
load.packages("MCMCglmm")
library(MCMCglmm)

k <- length(levels(data$4way.Category))
I <- diag(k-1)
J <- matrix(rep(1, (k-1)^2), c(k-1, k-1))

prior1 <-  list(R = list(fix = 1, V = 0.5 * (I + J), n = k - 1),
               G = list(G1 = list(V = diag(k-1), n = k-1, nu = k-1,
               alpha.mu = rep(0,3), alpha.V = diag(3)*a),
                       G2 = list(V = diag(2), n = 2, nu = 2,
                       alpha.mu = rep(0,2), alpha.V = diag(2)*a)))

model.table4 = MCMCglmm(QuestionType3 ~ -1 + trait + Group,
                    random = ~ us(trait):PartID + us(Group):PartID,
                    rcov = ~ us(trait):units,
                    prior = prior1,
                    thin = 50,
                    burnin = 32000,
                    nitt = 82000,
                    family = "categorical",
                    data = mcmc.Q3)
```

2 Correlation Analyses

This portion of the supplemental material provides a matrix of all the correlations that were conducted (in a heatmap format) and discussed the Results section.

Spearman rank correlations were computed to assess relationships between language and question outcome measures. The correlation matrix summarizes the correlations between the variables of interest (below). If there is a number reported in the matrix, this indicates a significant correlation; the darker the color the higher the correlation, with blue showing negative correlations and red showing positive correlations. The paper only reports significant findings, reporting on the semi-partial correlations that were conducted as follow ups to these correlations (partialing on the age of the child). Here, all significant and non-significant findings can be observed between the different measures.

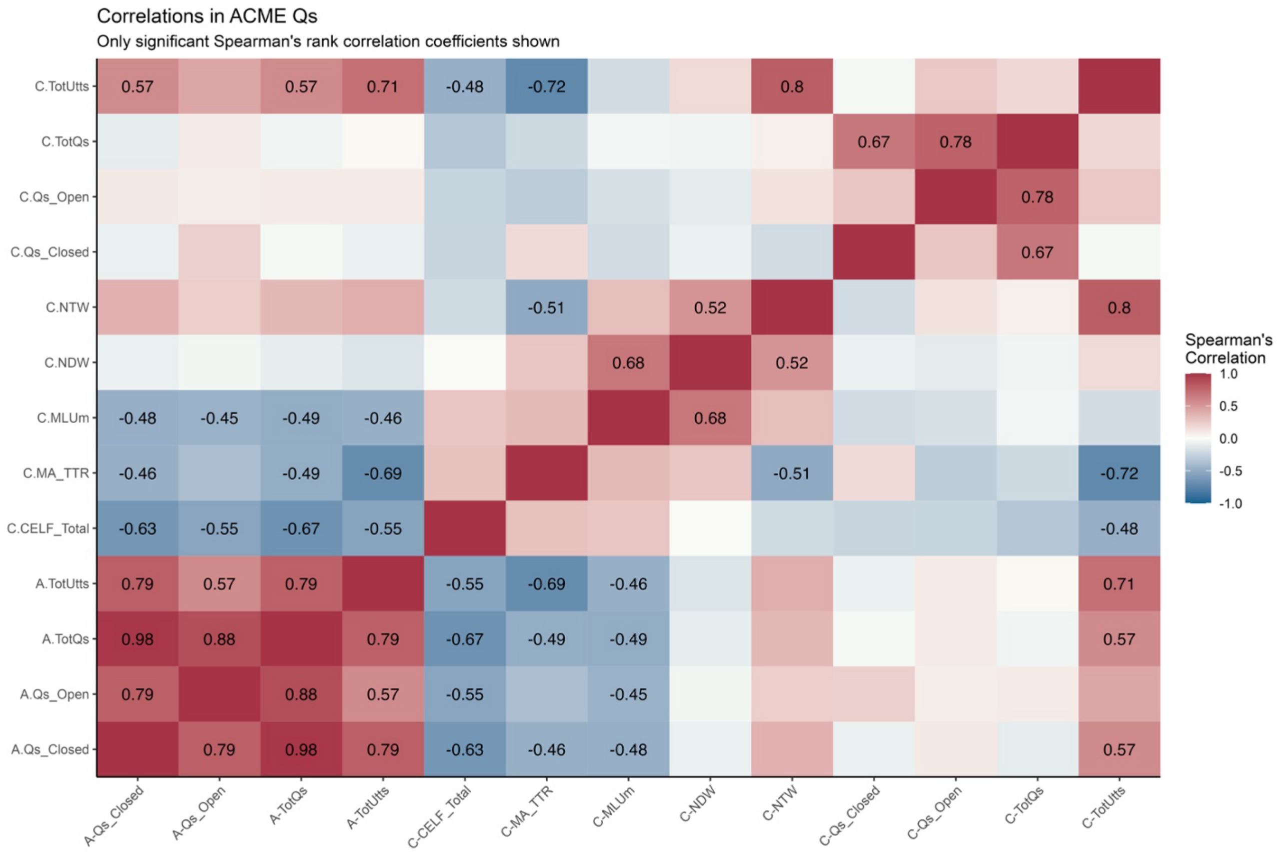

Supplementary Figure 1. Spearman rank correlation matrix for adult (A) and child (C) outcome measures of number of closed questions (Qs_Closed), number of open questions (Qs_Open), total number of questions (TotQs), total number of utterances (TotUtts), child CELF Core Language Score (CELF_Total), moving-average type-token ratio (MA_TTR), mean length utterance morpheme (MLUm), number of different words (NDW), and number of total words (NTW). Only significant Spearman's rank correlation coefficients are shown ($p < .05$); red indicates a positive correlation and blue a negative correlation.

3 Post Hoc Wh-Word Sub-Type Exploratory Analysis

This portion of the supplemental material provides a post hoc exploratory analysis of the breakdown of the different types of wh-word questions that were produced by the adults and children across both exhibits (see Supplementary Figure 2).

For adults and children, ‘what’ questions were the dominant type utilized, followed by ‘where’ and ‘how.’ Children also asked more ‘why’ questions than adult caregivers. The ‘where’, ‘who,’ and ‘when’ questions are typically narrower question types (i.e., more closed) and ‘how’ and ‘why’ are broader (i.e., more open). The ‘what’ question type can be of either category (broad or narrow) and an exploratory/impressionistic analysis of the category finds more broad/open usage than narrow/closed. Future work aims to include a more in-depth analysis to tease apart these details within the wh-question category.

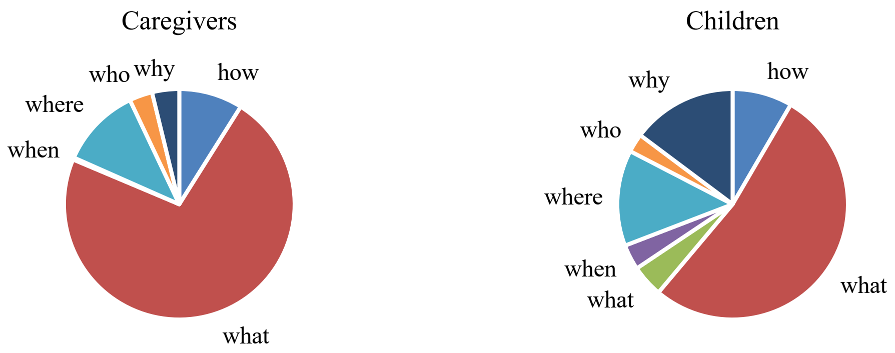

Supplementary Figure 2. Pie graphs depicting the percentage of wh-word question word sub-types used by adults and children across both exhibits.

4 Post Hoc Linear Regression Analysis

This portion of the supplemental material provides the output of a post hoc linear regression model to analyze the predictive power of child age on the adult proportional use of open-ended to closed questions (Supplementary Table 1). While assumptions were met to run the linear regression, the model was underpowered as the age groups each had a different number of participants and were unbalanced (ranging from 3 to 12 per group).

Here open-ended questions encompassed wh- (or content) questions and closed included polar, echo, and alternative questions. The model did not show any significant differences, though Supplementary Figure 3 shows a slight increasing trend (from ~.28 to .34) in the proportion of open-ended to closed questions used by caregivers of children aged three to five years old. The proportion used is similar for caregivers of children aged five and six years old.

Supplementary Table 1. Summary of linear regression results showing the coefficients, standard errors, t-values, and p-values for the predictor variable of child age group (3, 4, 5, and 6 years old) on the outcome variable of the proportion of caregiver open-ended to closed question types.

	Estimate	*SE*	*t*	*p*
(Intercept)	0.24	0.07	3.63	= 0.002
AgeGroup_Child	0.02	0.15	1.11	= 0.278

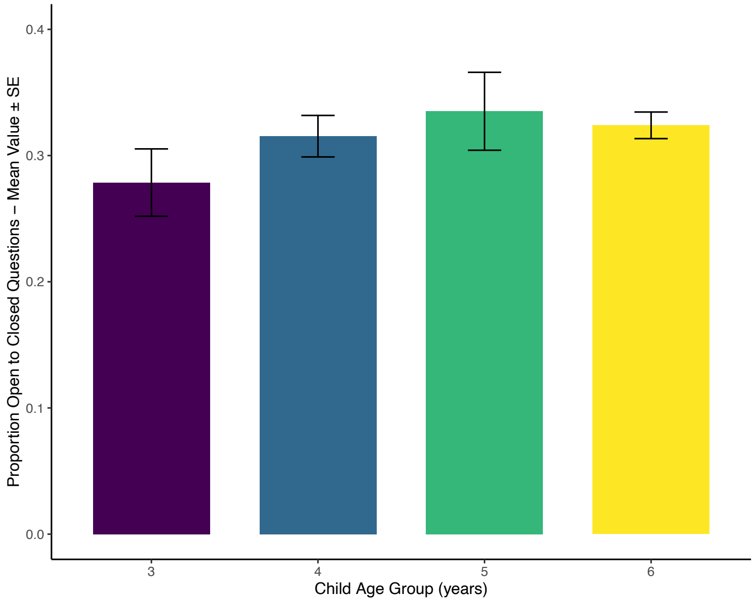

Supplementary Figure 3. Bar graph depicting caregiver mean proportions of open-ended to closed questions on the y-axis separated out by child age group (3, 4, 5, and 6 years old) on the x-axis. Error bars show standard error. Note that the sample sizes were unbalanced and differed by age group (from three up to twelve per group).
